# Supplementary material for: A Humanized Yeast Phenomic Model of Deoxycytidine Kinase to Predict Genetic Buffering of Nucleoside Analog Cytotoxicity
Source: Genes (Basel). 2019 Sep 30;10(10):770. doi: 10.3390/genes10100770 (PMC6826991; doi:10.3390/genes10100770)
Supplement: Supplementary file 1 [file genes-10-00770-s001.zip › Additional_Files/Additional_File7_GOTermHeatmaps.zip!/Additional_File7_GOTermHeatmaps/cytosolic_proteasome_complex.pdf]

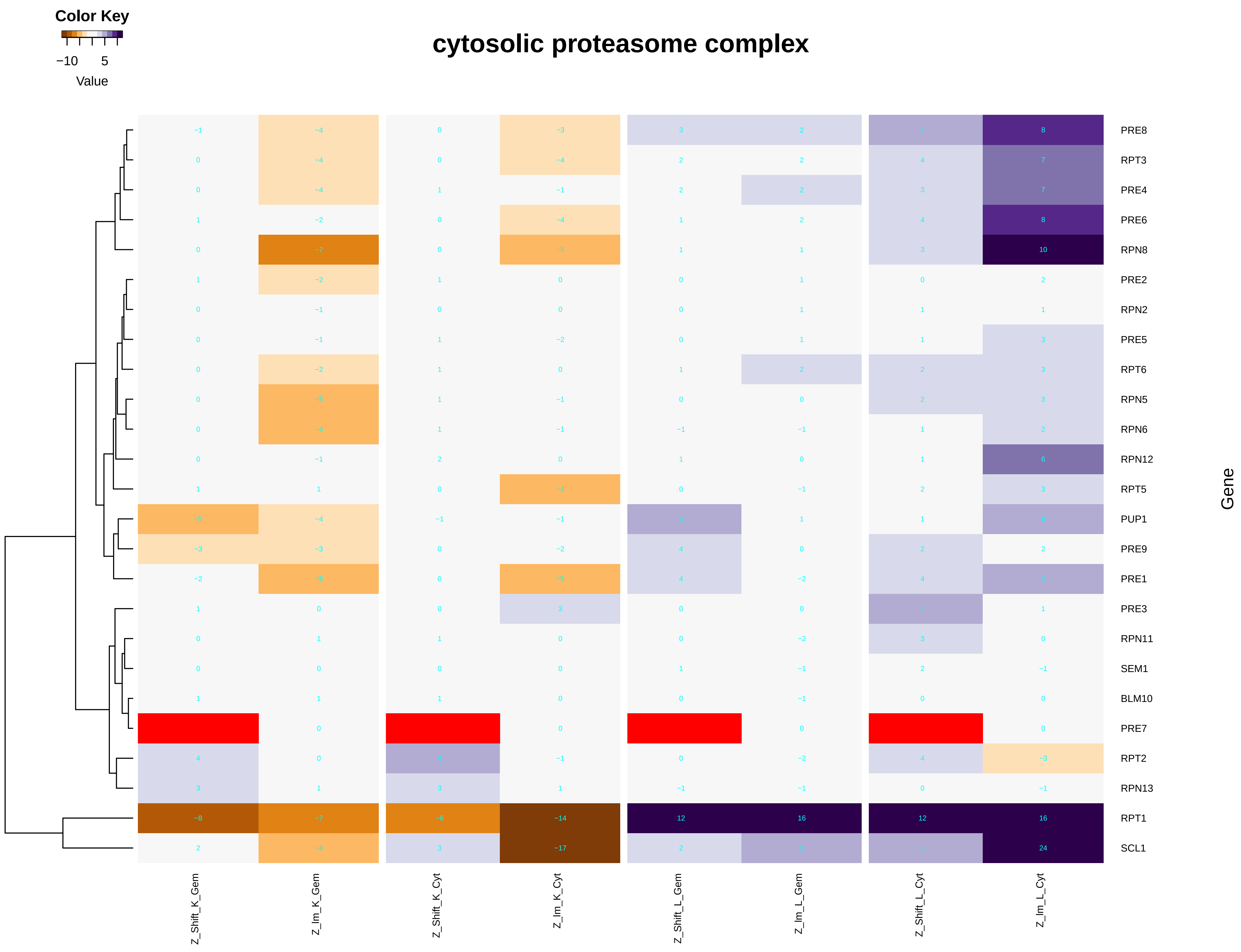
cytosolic proteasome complex
Color Key
−10
5
Value
Gene
PRE8
RPT3
PRE4
PRE6
RPN8
PRE2
RPN2
PRE5
RPT6
RPN5
RPN6
RPN12
RPT5
PUP1
PRE9
PRE1
PRE3
RPN11
SEM1
BLM10
PRE7
RPT2
RPN13
RPT1
SCL1
Z_Shift_K_Gem
Z_Im_K_Gem
Z_Shift_K_Cyt
Z_Im_K_Cyt
Z_Shift_L_Gem
Z_Im_L_Gem
Z_Shift_L_Cyt
Z_Im_L_Cyt

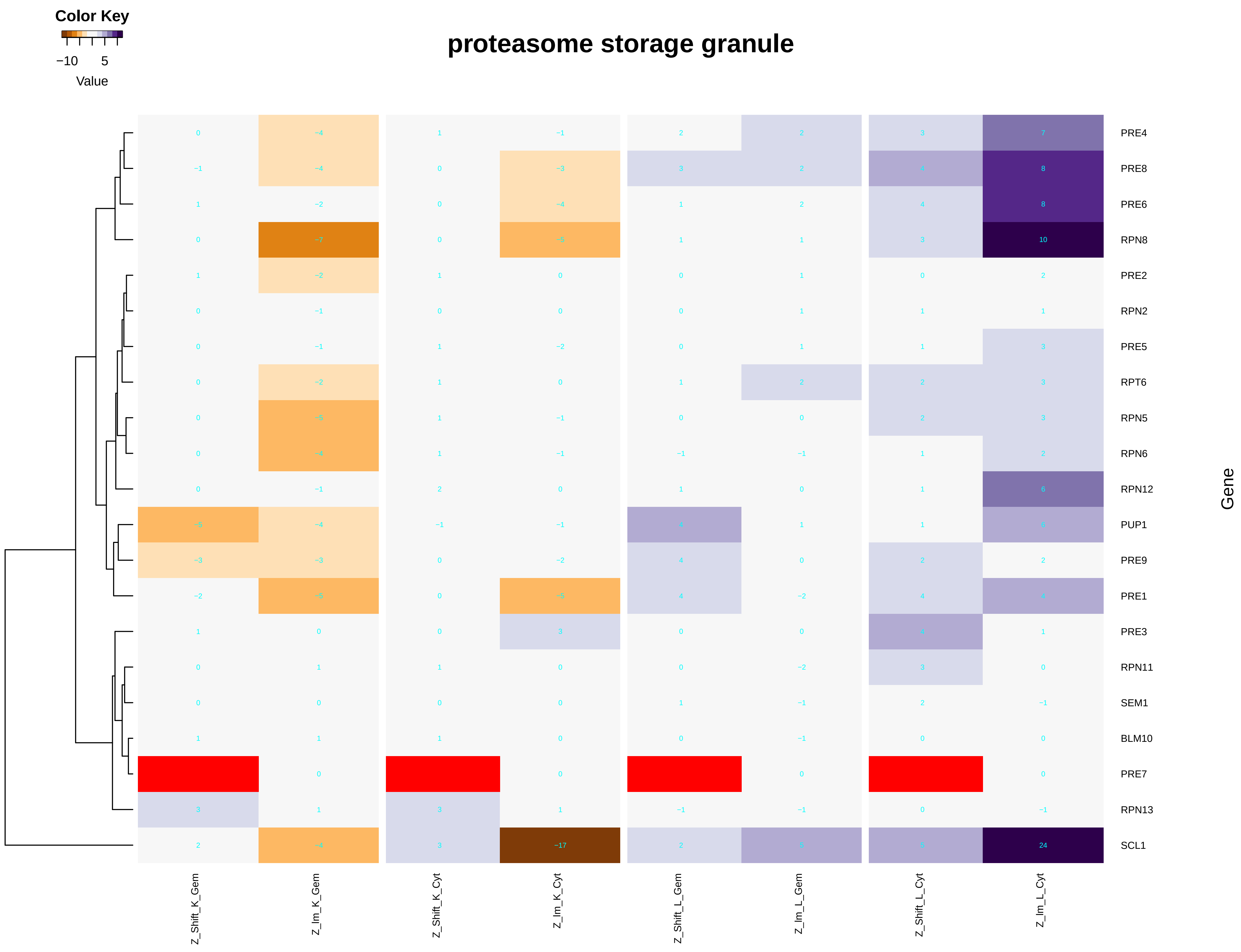
proteasome storage granule
Color Key
−10
5
Value
PRE4
PRE8
PRE6
RPN8
PRE2
RPN2
PRE5
RPT6
RPN5
RPN6
RPN12
PUP1
PRE9
PRE1
PRE3
RPN11
SEM1
BLM10
PRE7
RPN13
SCL1
Gene
Z_Shift_K_Gem
Z_Im_K_Gem
Z_Shift_K_Cyt
Z_Im_K_Cyt
Z_Shift_L_Gem
Z_Im_L_Gem
Z_Shift_L_Cyt
Z_Im_L_Cyt
